# Clinical Value of 18F-Fluorodeoxyglucose (FDG) Positron Emission Tomography (PET)/Computed Tomography (CT) in Radioiodine-Refractory Thyroid Carcinoma: A Case Report

**DOI:** 10.7759/cureus.90028

**Published:** 2025-08-13

**Authors:** David Gutiérrez Albenda, Laura N Rodríguez Varela, Julyana Murillo Jiménez, Paula Ulate Blanco, Juan J Solano Brenes

**Affiliations:** 1 Cyclotron-PET/CT Laboratory, University of Costa Rica, San José, CRI; 2 Medicine and Surgery, University of Costa Rica, San José, CRI

**Keywords:** 18f-fluorodeoxyglucose, differentiated thyroid cancer, papillary thyroid carcinoma, pet ct scan, thyroid radioiodine-refractory cancer

## Abstract

Radioiodine-refractory differentiated thyroid cancer is a condition that arises in patients who develop resistance to the mainstay treatment for thyroid cancer. Although it is relatively uncommon, its clinical significance lies in the fact that it markedly worsens prognosis and calls for the implementation of alternative management strategies for affected patients. This is a case of a 40-year-old woman with a 17-year history of differentiated thyroid cancer, refractory to radioiodine (RAI), showing no iodine-131 uptake on whole body scan scintigraphy and elevated tumor markers. Through 18F-fluorodeoxyglucose (18F-FDG) positron emission tomography (PET)/computed tomography (CT) imaging, hypermetabolic foci were identified in the lungs, consistent with metastatic recurrent disease. Due to its effectiveness, 18F-FDG PET/CT should be considered for evaluation in cases of clinical suspicion in patients with elevated tumor markers and low iodine-131 uptake, consistent with radioiodine-refractory disease.

## Introduction

Thyroid cancer is a common disease, and over the past few decades, its incidence has increased by up to 313%, mainly due to the development of diagnostic techniques. The term differentiated thyroid cancer (DTC) includes papillary thyroid carcinoma (PTC), which accounts for approximately 85% of cases, as well as other forms such as follicular and oncocytic carcinoma [1]. 

Despite its high incidence, the prognosis of DTC is generally favorable, as it is typically managed through therapeutic strategies including thyroidectomy, radioiodine ablation, and suppression of thyroid-stimulating hormone (TSH) levels. Nonetheless, a subset of patients exhibits a loss of iodine-131 (I-131) avidity, reflecting tumor dedifferentiation and indicating the development of radioiodine-refractory differentiated thyroid cancer (RAIR-DTC) [2]. Radioiodine refractoriness is an uncommon condition, with an estimated incidence of four to five cases per million patients annually. Among DTC, it is most frequently observed in poorly differentiated thyroid carcinoma and aggressive histological variants of papillary carcinoma. This condition markedly worsens prognosis, with a 10-year survival rate of less than 10%. [3,4].

Positron emission tomography (PET) has proven to be a valuable modality in the management of thyroid cancer, with 18F-fluorodeoxyglucose (18F-FDG) as the most widely utilized radioisotope. Moreover, the integration of concomitant computed tomography (CT) imaging has further supported its application for diagnostic, follow-up, and prognostic purposes [5,6].

The following clinical case illustrates the usefulness of PET/CT imaging with the radiotracer 18F-FDG in the evaluation of a possible case of RAIR-DTC.

## Case presentation

A 40-year-old female patient, with a prior history of acute lymphoblastic leukemia (ALL) diagnosed at the age of 11 and no history of radiotherapy as part of her leukemia management, presented with a well-differentiated PTC demonstrating an ongoing 17-year clinical course. The thyroid malignancy was initially identified in 2008, at the age of 28. During the initial phase of her therapeutic regimen, the patient underwent total thyroidectomy followed by ablative radioiodine therapy with I-131, which resulted in clinical remission.

During routine surveillance in 2019, a marked elevation in serum thyroglobulin (Tg) levels was detected, reaching 3,265 ng/mL in the absence of thyroid-stimulating hormone (TSH) stimulation. Table 1 illustrates the trend of Tg and TSH values over time. Due to the biochemical evidence of possible disease recurrence, the patient received an additional ablative dose of I-131, bringing the total cumulative dose at that time to 450 mCi. 

**Table 1:** Longitudinal behavior of biochemical markers. TSH: thyroid-stimulating hormone; Tg: thyroglobulin [7,8]

Year	2012	2019	2023	2025	Reference range
TSH (µIU/mL)	0.9	131	0.01	3	0.4-4.0
Tg (ng/mL)	40	3265	6.8	133	Suppressed Tg <0.2 or TSH-stimulated Tg <1 .0

Over the course of this period, the patient remained asymptomatic and under regular follow-up after neoplastic remission. In 2024, as part of scheduled monitoring, a chest CT scan revealed multiple pulmonary nodules suggestive of possible metastatic disease, with the surgical bed at the cervical level showing no abnormal contrast enhancement. Given these new findings indicative of potential recurrence, a diagnostic whole-body I-131 scan was performed, which reported no radioiodine uptake throughout the body. Due to the high clinical suspicion of cellular dedifferentiation, further evaluation with 18F-FDG PET-CT imaging was requested.

The patient was admitted in April 2025 with suspected pulmonary metastases based on conventional chest CT findings and an elevated Tg tumor marker level of 133 ng/mL. A high-resolution PET scan using lutetium-yttrium oxyorthosilicate (LYSO) detectors was performed, with axial plane acquisitions at a speed of 1 mm/s. This was combined with a low-dose CT scan (CareDOSE), performed using a 128-slice helical multidetector scanner with 5 mm slices and a pitch of 0.6 mm; the study was non-contrast-enhanced. The radiopharmaceutical used was 18F-FDG, administered intravenously via the right upper limb at a dose of 8.1 mCi. 

**Figure 1:**
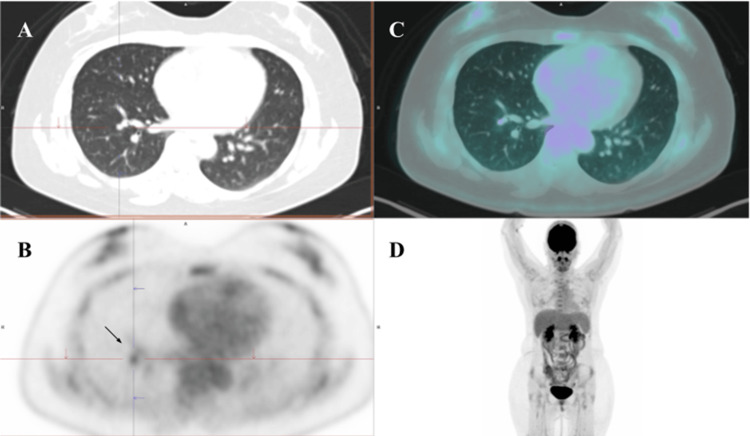
A) Axial CT (lung window): pulmonary lesion in the anterior basal segment of the right lower lobe. B) Axial PET: hypermetabolic focus in the anterior basal segment of the right lower lobe. C) Axial fused PET/CT image. D) Whole-body MIP image from FDG PET/CT. CT: computed tomography; PET: positron emission tomography; MIP: maximum intensity projection; FDG: fluorodeoxyglucose

**Figure 2:**
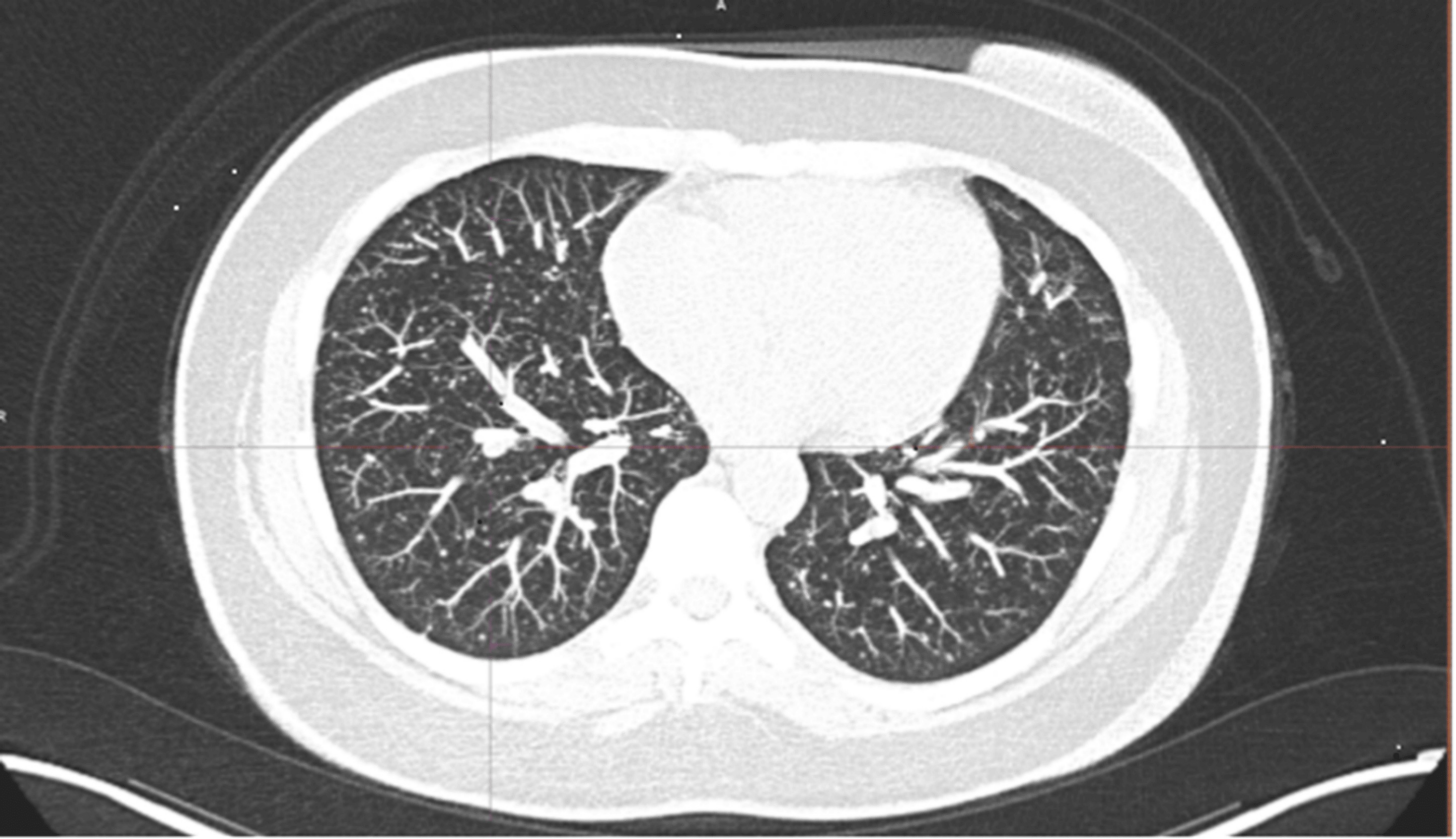
Axial CT slice (lung window), 10 mm MIP reconstruction, showing multiple bilateral pulmonary lesions. CT: computed tomography, MIP: maximum intensity projection.

The PET-CT scan revealed a non-calcified pulmonary nodule located in the anterior basal segment of the right lower lobe, with a peribronchial segmental distribution, measuring up to 6 mm (maximum standardized uptake value; Figure 1). Additionally, numerous bilateral pulmonary micronodules smaller than 4 mm were identified, randomly distributed and affecting both central and peripheral regions of the lung parenchyma. Some of these nodules were calcified, while others were non-calcified, probably below the metabolic resolution of the imaging modality (Figure 2). In this context, the combined findings of elevated tumor markers, a negative whole-body radioiodine scan, and positive uptake on PET/CT are consistent with metastatic disease from RAIR-DTC.

## Discussion

The first line of management of thyroid cancer begins with surgical resection of the primary tumor. According to the guidelines established by the American Thyroid Association (ATA), patients presenting with tumors larger than 4 cm, clinical evidence of regional lymph node metastases (N1), or distant metastases (M1), should undergo a total or near-total thyroidectomy as the initial surgical intervention to ensure complete excision of the original tumor. For neoplasms measuring between 1 cm and 4 cm, in the absence of metastatic spread or extrathyroidal extension, either a thyroid lobectomy or a near-total/total thyroidectomy may be considered as appropriate surgical options [7]. An important exception to immediate surgical intervention applies to patients diagnosed with unifocal papillary thyroid microcarcinomas (≤10 mm) without radiographic or clinical evidence of extracapsular extension or lymph node involvement. In such cases, active surveillance-including periodic ultrasound of the thyroid and cervical lymph nodes every six to 12 months is considered a safe and effective alternative to surgery [9].

Immediate postoperative management primarily aims to determine risk stratification for disease recurrence. The ATA has proposed a system to estimate the risk of recurrence, whereby the likelihood of thyroid cancer recurrence or persistence is determined by several factors, including tumor size, associated symptoms, macroscopic invasion of perithyroidal soft tissues, pathological N1 disease, extranodal extension, coexistence of B-Raf proto-oncogene (BRAF) V600E and telomerase reverse transcriptase (TERT) mutations, postoperative serum Tg levels suggestive of distant metastases, incomplete tumor resection, and presence of distant metastasis. The estimated risk of recurrence is stratified into three categories: low (≤5%), intermediate (6%-20%), and high (>20%) [7].

Radioiodine ablation therapy following total thyroidectomy is designed to eliminate any remaining normal thyroid tissue to ensure undetectable Tg levels, which subsequently serve as a marker for follow-up. This therapy also targets presumed foci of neoplastic cells as adjuvant treatment to reduce the risk of recurrence or to treat known persistent or recurrent disease. Doses of 30 mCi are typically prescribed for remnant ablation, whereas higher doses of 100-200 mCi are used for therapeutic purposes. For patients without known residual disease, management guidelines recommend treatment with doses greater than 100 mCi in those with intermediate to high risk of recurrence [3]. According to ATA guidelines, postoperative RAI ablation is not recommended for patients with low-risk differentiated thyroid cancer [7]. 

As demonstrated in the presented clinical case, some patients develop refractory disease despite timely surgical management and RAI therapy. In 2015, the ATA defined RAIR-DTC as a condition meeting any of the following criteria: 1. Complete absence of RAI uptake by malignant or metastatic tissue. 2. Loss of RAI avidity in tumor tissue that previously demonstrated the ability to concentrate radioactive iodine. 3. Presence of heterogeneous RAI avidity, with some lesions exhibiting uptake while others do not. 4. Disease progression to a metastatic state despite administration of RAI doses exceeding 600 millicuries (mCi) [7]. 

Despite this definition, a consensus among the ATA, the European Association of Nuclear Medicine (EANM), the Society of Nuclear Medicine and Molecular Imaging (SNMMI), and the European Thyroid Association (ETA) currently states that there is no single definition, clinical criterion, or classification system that serves as a definitive marker for identifying RAIR-DTC. Therefore, they recommend that each case should not be rigidly categorized by a single definition but rather evaluated in light of a spectrum of factors that influence the likelihood of a tumor being refractory to RAI [10].

At the molecular level, refractoriness has been associated with genetic and epigenetic alterations or disruptions in signaling pathways that result in decreased expression of the sodium-iodide symporter (NIS), diminished membrane targeting of NIS, or both [11]. Genetic alterations in the receptor tyrosine kinase (RTK)/(BRAF)/mitogen-activated protein kinase/extracellular signal-regulated kinase (MAPK/ERK) and phosphatidylinositol 3-kinase-protein kinase (PI3K) pathways have been identified as underlying causes of reduced NIS signaling, disabling iodine uptake and causing resistance to RAI therapy [12].

Neck ultrasound and serum thyroglobulin (Tg) measurements are central components of differentiated thyroid cancer follow-up. Monitoring Tg levels is an important method for detecting residual or recurrent disease following total or near-total thyroidectomy. An increase in Tg levels is strongly indicative of disease recurrence [13]. On the other hand, ultrasound is the most effective follow-up method for identifying structural disease in the neck, especially when residual thyroid tissue is present [3]. 

In instances of confirmed or suspected metastases, such as indicated by elevated serum Tg levels, further imaging investigations are warranted. Diagnostic whole-body radioiodine scintigraphy (WBS) may be employed for the detection of residual thyroid tissue or early identification of distant metastases; however, it has a low sensitivity ranging from 22% to 55%, but a high specificity between 90% and 100%. Despite this, false positives may occur. Its detection limits depend on the administered activity, the timing of image acquisition following RAI administration, the type of equipment used (crystal thickness), and the patient’s renal function status, as well as adequate patient preparation. Furthermore, the clinical utility of WBS is limited in patients whose tumors are no longer capable of concentrating RAI [6]. This may represent a case example consistent with the clinical presentation previously described.

The introduction of PET, based on the use of positron-emitting radionuclides and ring detectors to capture gamma photons generated by the annihilation of positrons with electrons, has emerged as an increasingly common option for the diagnosis and treatment of several types of cancer. In the field of oncological imaging, the most widely used radiotracer is the glucose analogue 18F-FDG, whose uptake reflects both normal and abnormal metabolic activity. The combination of PET with CT enhances the anatomical localization of lesions by integrating a functional study with a structural approach [14].

In the case of dedifferentiated thyroid cancer, two meta-analyses demonstrated that diagnostic sensitivity increases with the use of PET/CT for the detection of residual disease or recurrence in cases with elevated Tg levels and no evidence of RAI uptake [15,16]. For this reason, the ATA guidelines strongly recommend performing this study in high-risk patients with elevated Tg and negative RAI imaging [7].

Its application extends beyond diagnostic purposes, as it also leads to modifications in patient treatment strategies. It can be used as an initial staging tool in poorly differentiated thyroid cancer, as well as for post-treatment response assessment in cases of metastatic disease [7]. The uptake of 18F-FDG in patients with metastatic DTC is recognized as a prognostic factor for overall survival and serves as a major negative predictive marker for response to RAI therapy [6]. Accordingly, molecular imaging reflects the changes associated with RAI resistance and cellular dedifferentiation through a phenomenon known as "flip-flop," in which RAI uptake decreases while 18F-FDG uptake increases [17]. 

Although rare, the prognosis of patients with RAIR-DTC varies; however, their overall 10-year survival is less than 10%. Current management strategies for these patients include the use of tyrosine kinase inhibitors (TKIs), such as sorafenib and lenvatinib. In certain cases, local control of lesions through surgery or external beam radiation may also be considered. Additionally, other targeted systemic therapies are currently under investigation, aiming to improve outcomes in this challenging patient population [17]. 

## Conclusions

Radioiodine-refractory differentiated thyroid cancer, although uncommon, represents a clinically challenging condition associated with poor prognosis. Early recognition of these cases is essential for the selection and implementation of more effective therapeutic strategies. In this context, 18F-fluorodeoxyglucose (FDG) PET/CT has become a key tool not only for diagnosis but also for follow-up and prognostic evaluation in patients with RAI-refractory DTC. Integrating molecular imaging with emerging therapeutic advances may support the advancement of more personalized and effective approaches to management.
